# Short-term anti-plaque effect of a cymenol mouthwash analysed using the DenTiUS Deep Plaque software: a randomised clinical trial

**DOI:** 10.1186/s12903-023-03256-9

**Published:** 2023-08-12

**Authors:** B Suárez-Rodríguez, A Regueira-Iglesias, T Blanco-Pintos, C Balsa-Castro, N Vila-Blanco, MJ Carreira, I Tomás

**Affiliations:** 1 Oral Sciences Research Group, Special Needs Unit, Department of Surgery and Medical-Surgical Specialties, School of Medicine and Dentistry, Universidade de Santiago de Compostela, Health Research Institute of Santiago (IDIS), Santiago de Compostela, A Coruña 15782 Spain; 2 Centro Singular de Investigación en Tecnoloxías Intelixentes and Departamento de Electrónica e Computación, Universidade de Santiago de Compostela, Health Research Institute of Santiago (IDIS), Santiago de Compostela, A Coruña Spain

**Keywords:** Cymenol, Mouthwash, Dental plaque, Oral health, Prevention, Image analysis, Automated plaque quantification

## Abstract

**Background:**

The effect of cymenol mouthwashes on levels of dental plaque has not been evaluated thus far.

**Objective:**

To analyse the short-term, in situ, anti-plaque effect of a 0.1% cymenol mouthwash using the DenTiUS Deep Plaque software.

**Methods:**

Fifty orally healthy participants were distributed randomly into two groups: 24 received a cymenol mouthwash for eight days (test group A) and 26 a placebo mouthwash for four days and a cymenol mouthwash for a further four days thereafter (test group B). They were instructed not to perform other oral hygiene measures. On days 0, 4, and 8 of the experiment, a rinsing protocol for staining the dental plaque with sodium fluorescein was performed. Three intraoral photographs were taken per subject under ultraviolet light. The 504 images were analysed using the DenTiUS Deep Plaque software, and visible and total plaque indices were calculated (ClinicalTrials ID NCT05521230).

**Results:**

On day 4, the percentage area of visible plaque was significantly lower in test group A than in test group B (absolute = 35.31 ± 14.93% vs. 46.57 ± 18.92%, p = 0.023; relative = 29.80 ± 13.97% vs. 40.53 ± 18.48%, p = 0.024). In comparison with the placebo, the cymenol mouthwash was found to have reduced the growth rate of the area of visible plaque in the first four days by 26% (absolute) to 28% (relative). On day 8, the percentage areas of both the visible and total plaque were significantly lower in test group A than in test group B (visible absolute = 44.79 ± 15.77% vs. 65.12 ± 16.37%, p < 0.001; visible relative = 39.27 ± 14.33% vs. 59.24 ± 16.90%, p < 0.001; total = 65.17 ± 9.73% vs. 74.52 ± 13.55%, p = 0.007). Accounting for the growth rate with the placebo mouthwash on day 4, the above results imply that the cymenol mouthwash in the last four days of the trial reduced the growth rate of the area of visible plaque (absolute and relative) by 53% (test group A) and 29% (test group B), and of the area of total plaque by 48% (test group A) and 41% (test group B).

**Conclusions:**

The 0.1% cymenol mouthwash has a short-term anti-plaque effect in situ, strongly conditioning the rate of plaque growth, even in clinical situations with high levels of dental plaque accumulation.

## Introduction

Dental caries and periodontal diseases are among the most prevalent conditions globally, producing severe health and economic burdens that significantly reduce the quality of life of those affected [1]. Although these oral pathologies are multifactorial, dental biofilm plays a significant role in their initiation and development [2, 3].

There is consensus in the literature regarding the roles of professional tooth cleaning, oral hygiene instructions, and, especially, the self-performed mechanical removal of dental biofilm for the prevention and management of dental caries and periodontal diseases like gingivitis and periodontitis [2, 4]. However, in practice, most people fail to maintain an adequate level of plaque control since the effectiveness of brushing can be affected for reasons including: the time spent doing so, the difficulty in reaching the interproximal areas, poor dexterity, and a lack of adherence; all of which substantiate the need to employ complementary chemical hygiene methods [5, 6]. In this regard, using adjuvant chemical products in mouthwashes effectively remineralises decayed tissue [7, 8] and reduces gingival and bleeding indices in gingivitis patients [9]. Several studies have also demonstrated that these measures significantly affect the control of dental biofilm (from now on, dental plaque), thus preventing the development of the disease before its onset [9, 10].

Essential oils (EOs) are among the most-investigated active anti-plaque agents [11]. These complex products contain hundreds of chemical substances known for their anti-microbial, anti-inflammatory, or antioxidant properties [12]. The literature shows that using EO-containing rinses to complement oral hygiene measures improves oral health, mainly due to their anti-plaque and anti-gingivitis effects [13]. The gold standard agent against plaque and inflammation is chlorhexidine gluconate (CHX) [11]. Comparisons of CHX to EOs have shown that both components have an equivalent [14] or, in the case of EOs, even a superior impact on gingival indices [15]. In addition, CHX has been demonstrated in vitro to kill human gingival fibroblast cells faster and with more cytotoxic effects [16]. Moreover, CHX causes several adverse effects that do not occur with EOs, such as teeth staining, dry mouth, or taste disturbances [17]. Although reversible, they are uncomfortable during medium- to long-term treatment. Furthermore, as far as we are aware, EOs do not have one of the significant disadvantages associated with another widely studied anti-plaque and anti-gingivitis agent, cetylpyridinium chloride (CPC) [11], whose long-term use in low concentrations (such as in oral rinses) may carry a risk of causing anti-microbial resistance [18]. These factors make EOs an excellent alternative to CHX and CPC.

Within the family of EOs, o-cymen-5-ol (cymenol) is a natural phenolic compound derived from isopropyl cresol, whose mechanism of action is believed to be due to the alteration of the cell wall and cell membrane permeability [19, 20]. To date, only a few studies have conducted in vitro [21–24] or in vivo [25–31] experiments to assess the performance of this compound for distinct purposes.

Similar to the traditional approach employed in studies of other chemical adjuvants, the effects of cymenol on dental plaque levels have been evaluated via the Turesky clinical index [25, 26, 28, 32]. However, the inherent subjectivity of visual examinations, the laborious recording process, and the high degree of inaccuracy when plaque levels are too low or too high [33] may produce imprecise results and, as a consequence, complicate comparisons between chemical agents [34]. These factors have led to an exponential increase in using automated approaches to objectively quantify dental biofilm in recent years [35–37]. These tools include a clinically validated version developed by our research group: DenTiUS Deep Plaque software [37]. Nonetheless, to our knowledge, no study has used an automated image analysis method to evaluate the anti-plaque effects of cymenol.

Accordingly, the present investigation aimed to evaluate the short-term in situ anti-plaque effect of 0.10% cymenol mouthwashes using our DenTiUS Deep Plaque image analysis software.

## Materials and methods

This was a balanced, randomised, triple-blind, parallel study on the short-term anti-plaque effects of a commercialised cymenol-based mouthwash. The Ethics Committee of Clinical Investigation of Galicia (CEIC, Spain) approved the project and registered 2021/301. The protocol for this trial and the supporting CONSORT checklist are available as Supporting Material S1 and S2, respectively. The study was registered on ClinicalTrials.gov with the ID NCT05521230 (date of registration 30/08/2022). The authors can confirm that all ongoing and related trials for this intervention are recorded and can be accessed via the following URL: http://clinicaltrials.gov/ct2/show/NCT05521230. All the procedures conducted in the experiment were oral-based and explained in writing to all the participants. Written consent to participate in the project and to publish the results was obtained from the study participants. Publication of participant-identifiable data is not required; therefore, obtaining specific consent is not applicable.

### Selection of the study group: inclusion and exclusion criteria

Participants were sought for voluntary enrolment in the setting of the Faculty of Medicine and Dentistry of Santiago de Compostela (Universidade de Santiago de Compostela -USC-, Spain) from October 2021 to April 2022. Two clinicians adopting a previously standardised approach evaluated all the volunteers who verified compliance with the established inclusion and exclusion criteria. The subjects chosen were systemically healthy adults aged between 20 and 45 years with a good oral health status, i.e., a minimum of 24 permanent teeth, no evidence of gingivitis or periodontitis (bleeding on probing < 10%) [38], and an absence of untreated caries at the start of the study. The exclusion criteria were as follows: smoker or former smoker, the presence of dental prostheses or orthodontic devices, treatment with antibiotics or the routine use of oral antiseptics in the previous three months, and evidence of any systemic disease that could alter the production or composition of the saliva.

### Study phases and mouthwash protocols

**Fig. 1:**
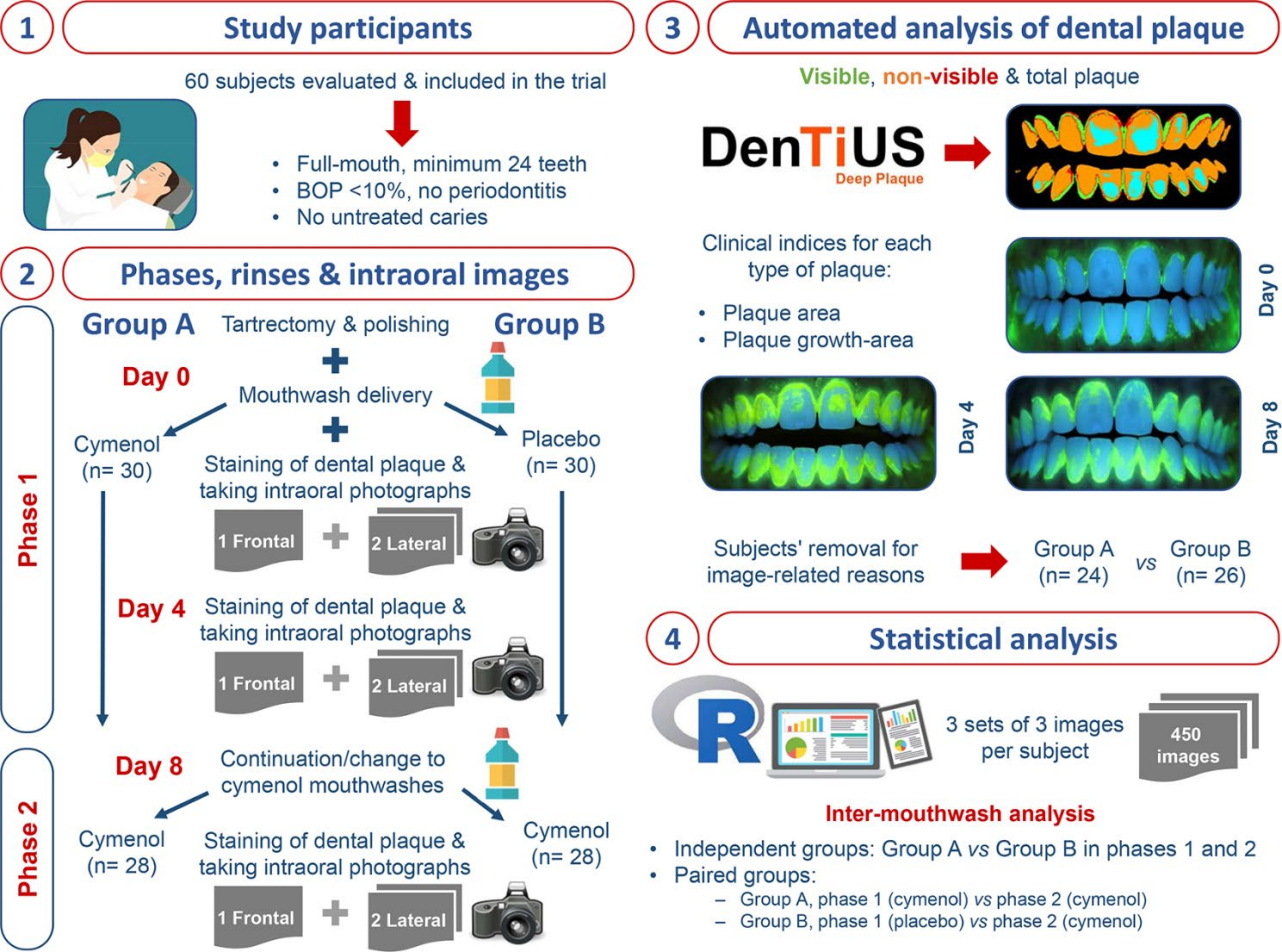
Flowchart of the development of this experiment

Adopting the above criteria, 60 participants were selected and randomly distributed into two groups. Two phases of mouthwash application were established (Fig. 1).

In phase 1, each group was randomly subjected to the application of mouthwash for four days: a cymenol mouthwash (test group A; n = 30), which corresponded to the product GingiLacer Encías Delicadas (active ingredients: 0.10% cymenol, 0.10% zinc chloride, potassium glycyrrhizate, and fluoride salts); or a placebo mouthwash (test group B; n = 30), which contained no active ingredients but was organoleptically similar or identical to the cymenol version. The participants used a rinse of 10 millilitres (ml) for 60 s, three times a day, with an interval of seven to nine hours between them. Then, in phase 2, all the participants used the cymenol mouthwash for four days following the exact dosing and schedule as in phase 1.

The volunteers performed no other oral hygiene measures during the eight days of mouthwash application. To evaluate their compliance, the antiseptic bottles were weighed at the beginning and end of the two application phases. Each mouthwash (cymenol and placebo) was provided to a participant in an opaque bottle with instructions about the necessary volume and an extra amount for possible losses. The R free distribution software [39] was employed to conduct a balanced randomisation process for allocating the mouthwashes, and the designation list was recorded in an Excel file.

### Staining of dental plaque and intraoral photography

The participants attended the Faculty of Dentistry on two occasions during the development of phase 1 and once in phase 2. In the first appointment for phase 1 (day 0), we performed ultrasound scaling and polishing with a brush/cup and polishing pastes. Dental floss or interproximal brushes were used where appropriate for the interproximal areas. In this way, all the participants had an initial level of dental plaque close to zero at the start of the experiment. A series of rinses was then employed to stain the dental plaque, with sodium fluorescein as the developer:

- One rinse for 10 s with 20 ml of phosphate buffer.
- One rinse for one minute with 15 ml of 1240 parts per million (ppm) fluorescein in a phosphate buffer.
- Three rinses for 10 s with 25 ml of phosphate buffer.

At both the second appointment in phase 1 (day 4) and the only one in phase 2 (day 8), the same rinsing protocol as that described above was performed to stain the dental plaque with sodium fluorescein.

On days 0, 4, and 8, the participants in each mouthwash test group (i.e., test group A or B) performed the sodium fluorescein rinse protocol before intraoral photographs were taken under ultraviolet light using our self-designed intraoral image replication device (number of patent registration: 2572333). This has been designed based on the following requirements that guarantee the positional standardisation of all its elements: the rigidity and stability of the device as a whole; the option to adjust and spatially fix the attachments to enable it to be adapted to any individual; the capacity to register its position numerically; and the ease of handling and transport.

**Fig. 2:**
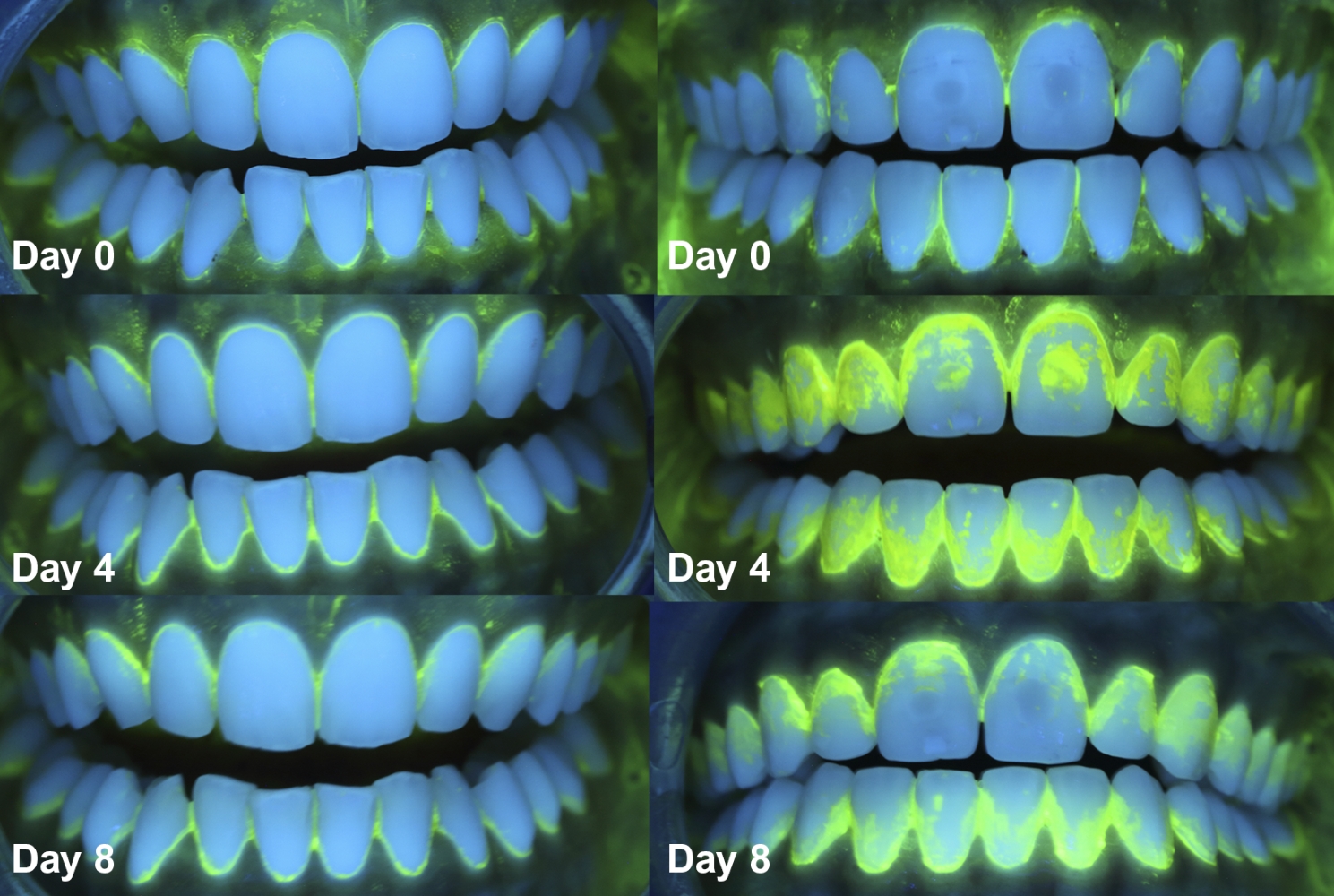
Photographs were taken under ultraviolet light on days 0, 4, and 8 in one patient of test group A (left) and one patient of test group B (right)

Three intraoral photographs were taken of each subject at each appointment: one frontal (from canine to canine) and two lateral (from the premolar to the first molar, including the upper and lower sectors) (Fig. 2). The photographs were taken with an exposure time of 1” and reviewed immediately after capture; a second image was obtained if the operator considered it appropriate.

### Analysis of the intraoral photographs using the DenTiUS Deep Plaque software

The digital quantification of the bacterial plaque in the photographic images was carried out using an image processing program of our design named DenTiUS Deep Plaque [37]. This was developed by USC’s Oral Sciences Research Group (OSRG) and the Centro Singular de Investigación en Tecnoloxías Intelixentes (CiTIUS).

**Fig. 3:**
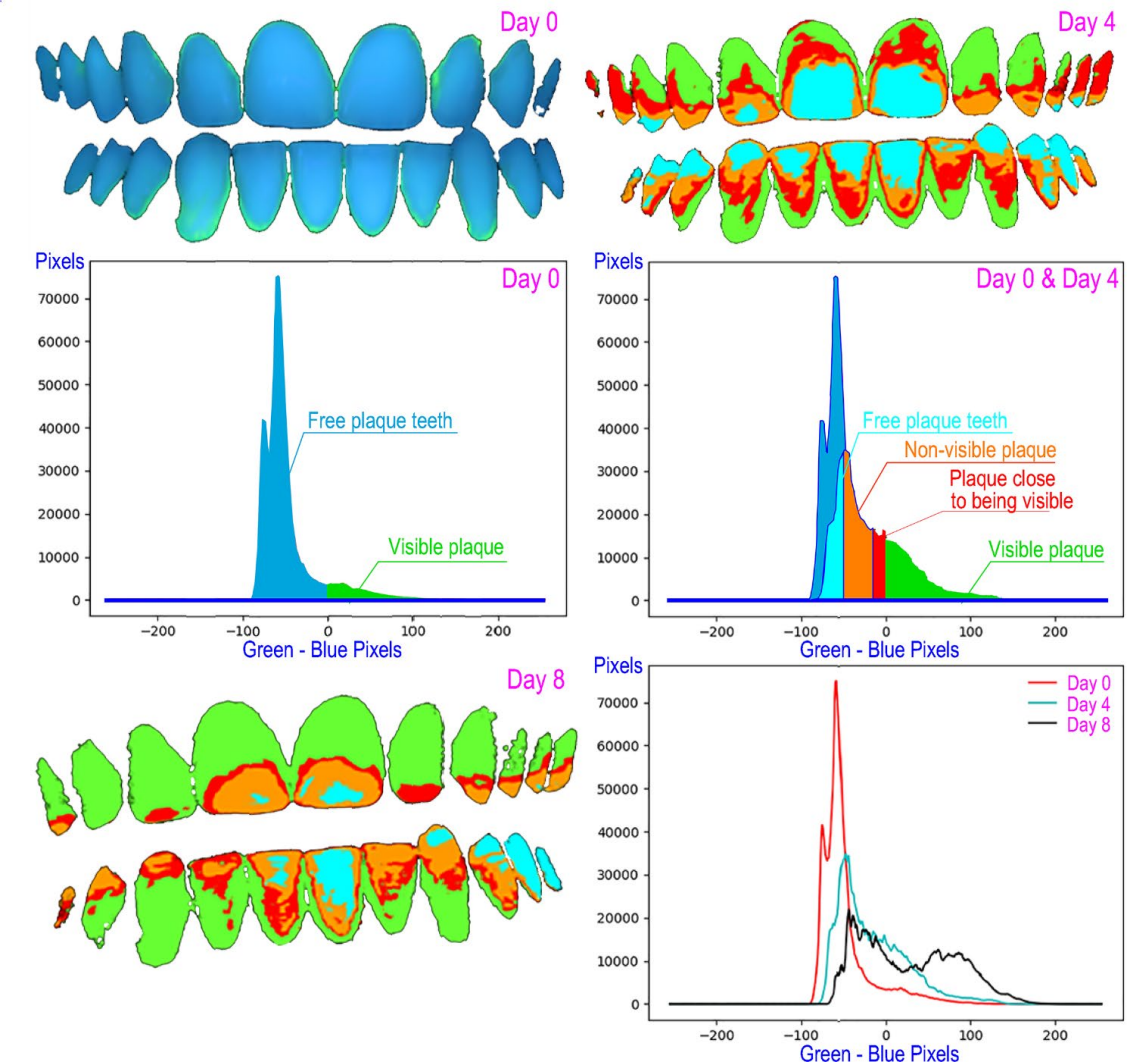
Determination of dental plaque levels by DenTiUS Deep Plaque software: graphical representation of plaque-free teeth, visible plaque, and non-visible plaque

DenTiUS Deep Plaque is an application based on digitally processing photographic images of dentition to quantify the bacterial plaque on tooth surfaces. Its use is indicated for analysing dental plaque evolution patterns and evaluating the effectiveness of different oral hygiene measures. The software allows the automatic determination of the levels of visible, non-visible, and total dental plaque (Fig. 3). The visible plaque is defined as a plaque with a green channel pixel value higher than the blue channel value. The non-visible plaque is defined as a plaque whose green channel pixel value is higher than that of a reference time (professional dental cleaning) but is not higher than the blue channel value, although both values are close. Lastly, the sum of the two plaques is known as the total plaque [37].

A series of clinical indices can be calculated for each type of plaque, and the following are those that were evaluated in the present trial:

- Plaque area: Percentage of the tooth surface with dental plaque.
- Plaque growth area: The growth rate of the dental plaque area per unit of time in hours (percentage area/hour).

**Fig. 4:**
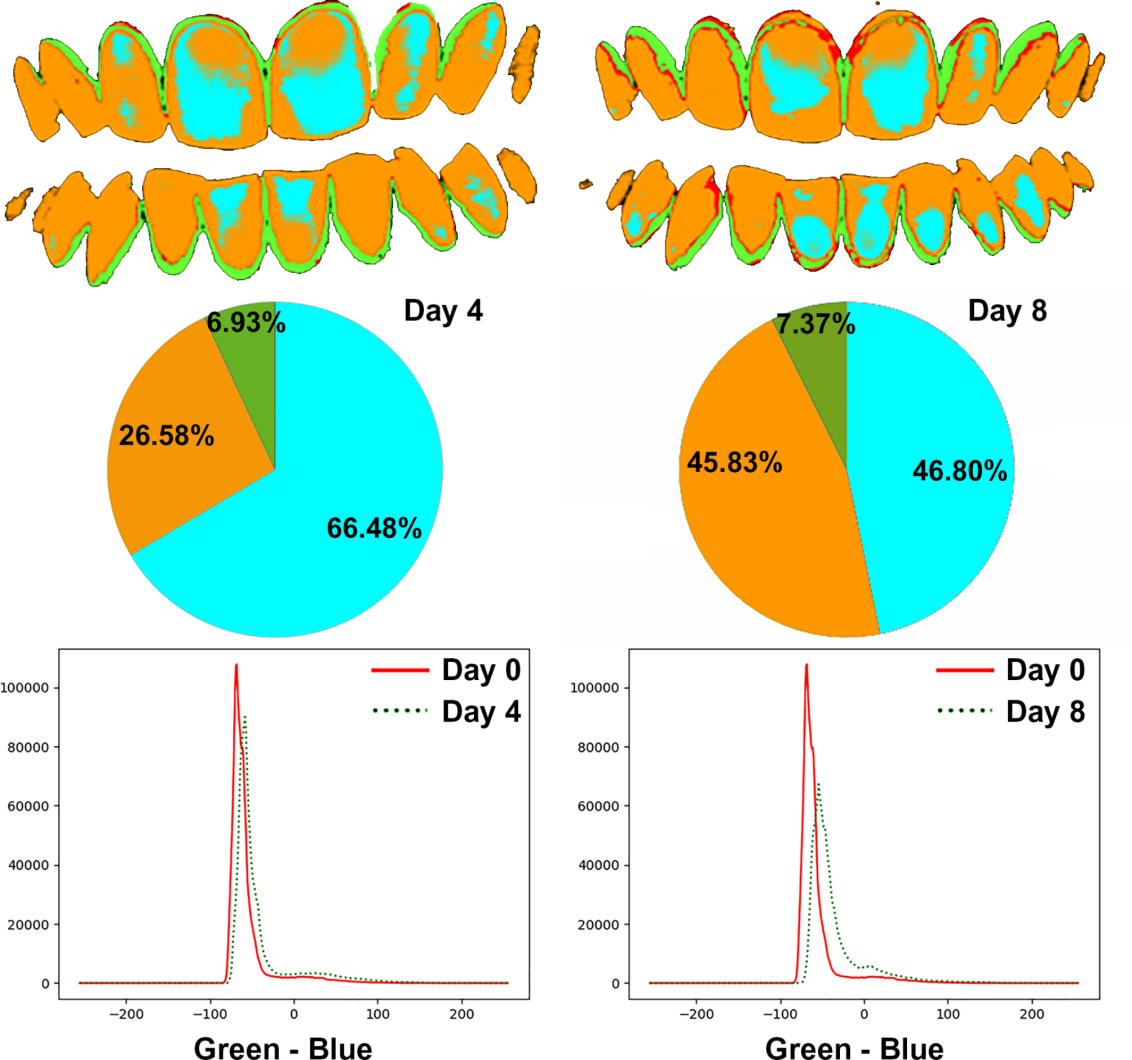
Quantification of visible plaque and non-visible plaque levels on day 4 (left) and day 8 (right) in an example patient in test group A

**Fig. 5:**
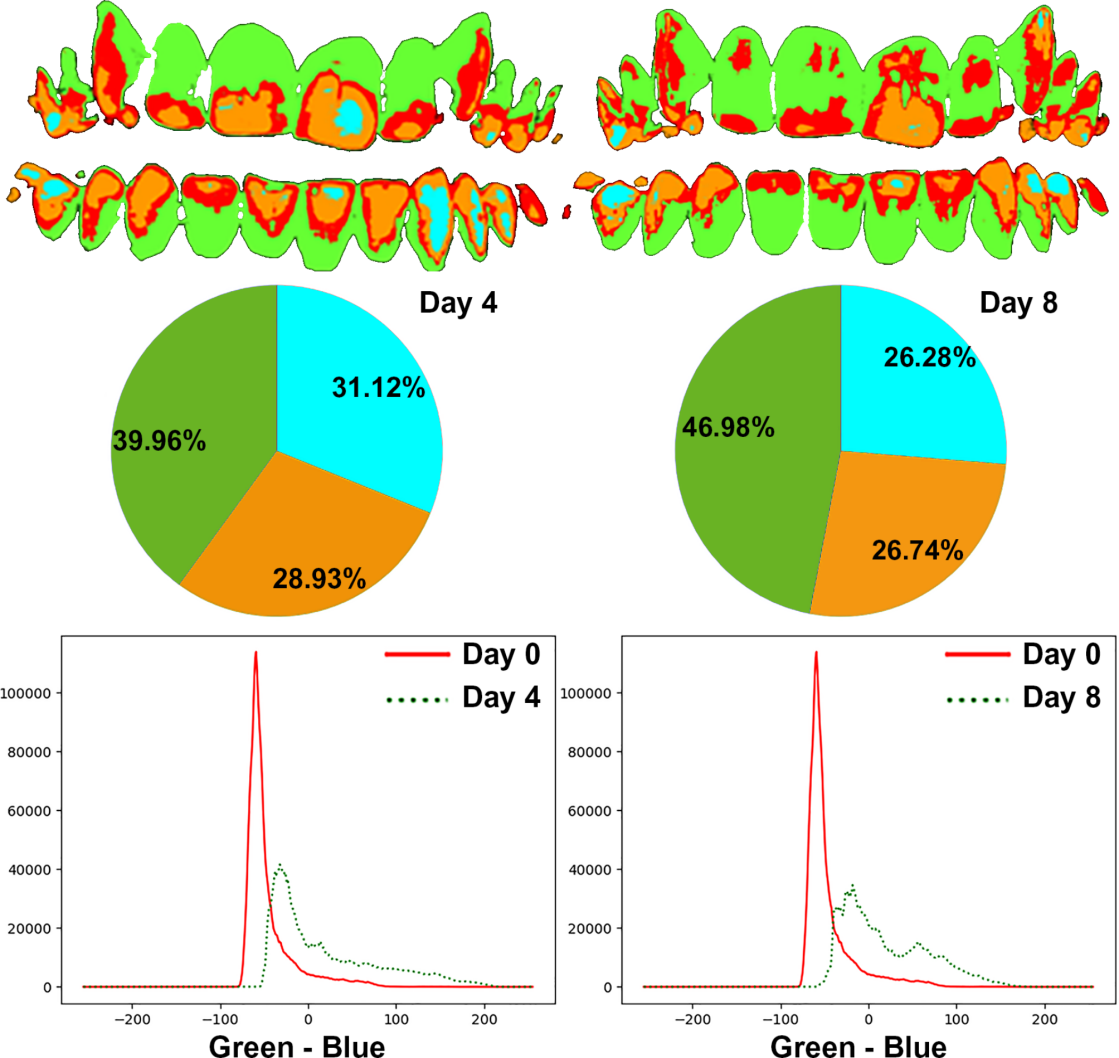
Quantification of visible plaque and non-visible plaque levels on day 4 (left) and day 8 (right) in an example patient in test group B

These indices were calculated absolutely on the analysed image without using a previous reference and relatively on the analysed image utilising the moment of professional dental cleaning as a reference (Figs. 4 and 5).

### Statistical analysis

Given that the present study had a parallel design of independent groups, and accounting for the possibility of using a two-tailed test of mean differences between two independent groups, an effect size of 0.80, an alpha error of 0.05, and a statistical power of 0.80, a minimum sample size of 26 subjects were required in each group. Due to the possible loss of patients for various reasons, each study group initially consisted of 30 participants. The sample size calculation was performed with the program G*Power version 3.1.9.4 [40].

The statistical analysis was conducted using the freely distributed R software [39]. In this clinical trial, the unit of analysis was the set of intraoral photographs obtained at each time point (days 0, 4, and 8) in the two phases of mouthwash application. The initial sample size in the investigation was 60 patients, four of whom were excluded for failing to participate in both study phases, five for poor quality photographs or inadequate fluorescein performance, and one for improper baseline plaque levels. The final sample size was, therefore, 50 patients; for each of them, three sets of three intraoral photographs (one frontal and two lateral) were taken to determine their levels of dental plaque. Consequently, 150 sets consisting of a total of 450 intraoral photographs were evaluated. Two differentiated types of statistical analyses were performed:

- Inter-mouthwash analysis between independent groups: test group A vs. test group B in phase 1 (cymenol and placebo mouthwashes, respectively); test group A vs. test group B in phase 2 (cymenol mouthwashes in both groups). After using the Shapiro-Wilk test to determine the normal distribution of the data, either the Student’s t-test for independent samples (normal distribution) or the Mann-Whitney U (non-normal distribution) test was employed to compare the means obtained in both groups with the DenTiUS Deep Plaque clinical indices at baseline (day 0) and after using the mouthwash (days 4 and 8). In all cases, statistical significance was set as *p* < 0.05.
- Inter-mouthwash analysis in a paired group: test group A in phase 1 (cymenol mouthwash) vs. phase 2 (cymenol mouthwash), and test group B in phase 1 (placebo mouthwash) vs. phase 2 (cymenol mouthwash). After testing for the normal distribution of the data using the Shapiro-Wilk test, either the repeated measures ANOVA (normal distribution) or the Wilcoxon test (non-normal distribution) and post hoc tests were applied with the Bonferroni correction to obtain a pairwise comparison of the DenTiUS Deep Plaque (DDP) clinical indices for a particular study group at the different timepoints (days 0, 4, and 8). In all cases, statistical significance was set as a *p*-corrected value < 0.016.

## Results

### Clinical characteristics of the study groups

Ten volunteers were excluded from the initial sample of 60 participants, leaving 50 subjects separated into two study groups: test group A (n = 24) and test group B (n = 26). The mean ages of the volunteers were 21.87 ± 1.51 and 22.00 ± 3.07, respectively, with a predominance of females in both groups (58.33% and 76.92%, respectively). In the whole-mouth assessment, all the participants had very low levels of bacterial plaque (mean = 6%) and periodontal parameters indicative of health (mean gingival bleeding = 3%, probing depth = 1.75 mm, and absence of clinical attachment loss). No significant differences were detected between the two study groups in any of the clinical parameters recorded (Table 1).

**Table 1:** Clinical characteristics of the study groups

Clinical parameters registered	Test group A (n = 24)	Test group B (n = 26)	p-value
Age (years)	21.87 ± 1.51	22.00 ± 3.07	0.835
Gender			
Men	10	6	
Women	14	20	0.227
Number of teeth	28.54 ± 1.69	29.03 ± 1.56	0.284
Bacterial plaque (%)	6.20 ± 6.00	6.40 ± 6.20	0.914
Gingival bleeding (%)	3.20 ± 3.00	3.40 ± 3.20	0.821
Probing pocket depth (mm)	1.79 ± 0.22	1.70 ± 0.24	0.184

Values indicate means (± standard deviations) and the number of subjects. After applying the Shapiro-Wilk test and determining the non-normal distribution of almost all the clinical variables, the Mann-Whitney U test was used to compare the quantitative clinical variables between the two study groups; the exception was the variable “probing pocket depth” (where the Student’s t-test was applied for independent groups). Fisher´s exact test was used to assess the association of the qualitative variables between the two study groups. A significance level of p < 0.05 was established

mm, millimetres; SD, standard deviation

### Inter-mouthwash analysis between independent groups: test group A *vs.* test group B in phases 1 and 2

Table 2 compares the DenTiUS Deep Plaque clinical indices obtained for each group in phase 1 (days 0 and 4) and phase 2 (day 8).

**Table 2:** DenTiUS Deep Plaque clinical indices were obtained in test groups A and B in phase 1 (after using the cymenol and placebo mouthwashes, respectively) and phase 2 (after using the cymenol mouthwash in both groups)

DenTiUS Deep Plaque clinical indices	Test group A (Mean % ± S.D.)	Test group B (Mean % ± S.D.)	*p*-value
**Phase 1_Day 0**			
Visible plaque area (absolute)	5.57 ± 3.57	5.68 ± 3.53	0.906
**Phase 1_Day 4**			
Visible plaque area (absolute)	35.31 ± 14.93	46.57 ± 18.92	0.023
Visible plaque area (relative)	29.80 ± 13.97	40.53 ± 18.48	0.024
Total plaque area	57.49 ± 13.26	62.75 ± 16.85	0.225
Visible plaque area growth (absolute)	0.36 ± 0.15	0.49 ± 0.20	0.016
Visible plaque area growth (relative)	0.31 ± 0.14	0.43 ± 0.19	0.017
Total plaque area growth	0.60 ± 0.13	0.66 ± 0.17	0.150
**Phase 2_Day 8**			
Visible plaque area (absolute)	44.79 ± 15.77	65.12 ± 16.37	< 0.001
Visible plaque area (relative)	39.27 ± 14.33	59.24 ± 16.90	< 0.001
Total plaque area	65.17 ± 9.73	74.52 ± 13.55	0.007*
Visible plaque area growth (absolute)	0.23 ± 0.08	0.34 ± 0.08	< 0.001
Visible plaque area growth (relative)	0.20 ± 0.07	0.31 ± 0.08	< 0.001
Total plaque area growth	0.34 ± 0.05	0.39 ± 0.07	0.005*

Total plaque: visible plaque + non-visible plaque

SD, standard deviation

*Due to the non-normal distribution of some of the contrasted variables, the Mann-Whitney U test was applied to compare the two groups

In phase 1 on day 0 (after professional dental cleaning), the percentages of the visible plaque areas were similar between the groups (5.57 ± 3.57% vs. 5.68 ± 3.53%, p = 0.906).

On day 4 of the same phase, the percentage of the visible plaque area was significantly lower in the cymenol subjects than in the placebo subjects, both in absolute (35.31 ± 14.93% vs. 46.57 ± 18.92%, p = 0.023) and relative (29.80 ± 13.97% vs. 40.53 ± 18.48%, p = 0.024) terms. The subjects who used the cymenol mouthwashes also had a lower total plaque area value (57.49 ± 13.26% vs. 62.75 ± 16.85% in the placebo subjects, p = 0.225), although these results did not achieve statistical significance. During the first four days of the experiment, the visible plaque area growth rate was significantly lower in the cymenol participants than in those in the placebo participants, both in absolute (0.36 ± 0.15% vs. 0.49 ± 0.20%, p = 0.016) and relative (0.31 ± 0.14% vs. 0.43 ± 0.19%, p = 0.017) terms. Concerning the placebo, these results mean that the cymenol mouthwash reduced the growth rate of the visible plaque area in the first four days by 26% (absolute) to 28% (relative).

Again, in phase 2 on day 8, the percentage of the visible plaque area was significantly lower in test group A than in test group B (absolute = 44.79 ± 15.77% vs. 65.12 ± 16.37%, p < 0.001; relative = 39.27 ± 14.33% vs. 59.24 ± 16.90%, p < 0.001), as was the percentage of the total plaque area (65.17 ± 9.73% vs. 74.52 ± 13.55%, p = 0.007). Despite both groups using the cymenol mouthwash in the last four days of the trial, the visible plaque area growth rate was significantly lower in the cymenol volunteers than those given the placebo (absolute = 0.23 ± 0.08 vs. 0.34 ± 0.08, p < 0.001; relative = 0.20 ± 0.07 vs. 0.31 ± 0.08, p < 0.001); this was also the case for the total plaque area growth rate (0.34 ± 0.05 vs. 0.39 ± 0.07, p = 0.005). However, given the growth that occurred with the placebo mouthwash on day 4, the results above imply that the use of the cymenol mouthwash in the last four days of the trial reduced the growth rate of both the visible plaque area (absolute and relative) in these four days by 53% (test group A) and 29% (test group B), and the total plaque area by 48% (test group A) and 41% (test group B).

### Inter-mouthwash analysis in a paired group: test group A, phase 1 (cymenol mouthwash) *vs.* phase 2 (cymenol mouthwash)

In test group A, which received the cymenol mouthwash in both phase 1 and phase 2, there were statistically significant differences in the percentages of the visible plaque area between day 0 (5.57 ± 3.57%) vs. day 4 (35.31 ± 14.93%, p < 0.001) and day 8 (44.79 ± 15.77%, p < 0.001).

Table 3 compares the DenTiUS Deep Plaque clinical indices obtained for this group on day 4 in phase 1 vs. day 8 in phase 2. The visible and total plaque areas were significantly increased on day 8 compared to day 4 (absolute = 44.79 ± 15.77% vs. 35.31 ± 14.93%, p = 0.001; relative = 39.27 ± 14.33% vs. 29.80 ± 13.97%, p = 0.001; total = 65.17 ± 9.73% vs. 57.49 ± 13.26%, p = 0.004). However, despite the progressive increase in plaque levels throughout the trial, the rate of plaque growth was significantly lower with the use of the cymenol treatment over the last four days (day 8 vs. day 4: absolute = 0.23 ± 0.08% vs. 0.36 ± 0.15%, p < 0.001; relative = 0.20 ± 0.07% vs. 0.31 ± 0.14%, p < 0.001; total = 0.34 ± 0.05% vs. 0.60 ± 0.13%, p < 0.001). These results mean that, concerning day 4, the cymenol mouthwashes reduced the growth rate of the visible plaque area in the last four of the eight days by 35% (relative) to 36% (absolute), and the total plaque area growth rate by 43%.

**Table 3:** DenTiUS Deep Plaque clinical indices were obtained for test group A on day 4 (phase 1: receiving the cymenol mouthwash) and day 8 (phase 2: receiving the cymenol mouthwash)

DenTiUS Deep Plaque clinical indices	Test group A		*p*-value
	Phase 1_Day 4 (Mean % ± S.D.)	Phase 2_Day 8 (Mean % ± S.D.)	
Visible plaque area (absolute)	35.31 ± 14.93	44.79 ± 15.77	0.001
Visible plaque area (relative)	29.80 ± 13.97	39.27 ± 14.33	0.001
Non-visible plaque area	27.69 ± 11.27	25.89 ± 10.37	0.262
Total plaque area	57.49 ± 13.26	65.17 ± 9.73	0.004*
Visible plaque area growth (absolute)	0.36 ± 0.15	0.23 ± 0.08	< 0.001
Visible plaque area growth (relative)	0.31 ± 0.14	0.20 ± 0.07	< 0.001
Total plaque area growth	0.60 ± 0.13	0.34 ± 0.05	< 0.001*

Total plaque: visible plaque + non-visible plaque

SD, standard deviation

*Due to the non-normal distribution of some of the contrasted variables, the Wilcoxon test was applied to compare the two groups

### Inter-mouthwash analysis in a paired group: test group B, phase 1 (placebo mouthwash) *vs.* phase 2 (cymenol mouthwash)

In test group B, which received the cymenol mouthwash in phase 2, there were statistically significant differences in the percentages of the visible plaque area between day 0 (5.68 ± 3.53%) vs. day 4 (i.e., after the placebo mouthwash: 46.57 ± 18.92%, p < 0.001) and day 8 (i.e., after the cymenol mouthwash; 65.12 ± 16.37%, p < 0.001).

Table 4 compares the DenTiUS Deep Plaque clinical indices obtained for this group on day 4 in phase 1 vs. day 8 in phase 2. This demonstrates how the percentages of the areas of visible and total plaque increased significantly on day 8 compared to day 4 (absolute = 65.12 ± 16.37% vs. 46.57 ± 18.92%, p < 0.001; relative = 59.24 ± 16.90% vs. 40.53 ± 18.48%, p < 0.001; total = 74.52 ± 13.55% vs. 62.75 ± 16.85%, p < 0.001). However, despite this progressive increase in plaque levels, the rate of plaque growth was significantly lower with the application of the cymenol mouthwash over the last four of the eight days (day 8 vs. day 4: absolute = 0.34 ± 0.08% vs. 0.49 ± 0.20%, p < 0.001; relative = 0.31 ± 0.08% vs. 0.43 ± 0.19%, p = 0.001; total = 0.39 ± 0.07% vs. 0.67 ± 0.18%, p < 0.001). Accordingly, concerning day 4, the cymenol mouthwashes reduced the growth rate of the visible plaque area in the last four days by 28% (relative) to 30% (absolute) and the growth rate of the total plaque area by 41%.

**Table 4:** DenTiUS Deep Plaque clinical indices were obtained for test group B on day 4 (phase 1: receiving the placebo mouthwash) and day 8 (phase 2: receiving the cymenol mouthwash)

DenTiUS Deep Plaque clinical indices	Test group B		*p*-value
	Phase 1_Day 4 (Mean % ± S.D.)	Phase 2_Day 8 (Mean % ± S.D.)	
Visible plaque area (absolute)	46.57 ± 18.92	65.12 ± 16.37	< 0.001
Visible plaque area (relative)	40.53 ± 18.48	59.24 ± 16.90	< 0.001
Total plaque area	62.75 ± 16.85	74.52 ± 13.55	< 0.001
Visible plaque area growth (absolute)	0.49 ± 0.20	0.34 ± 0.08	< 0.001
Visible plaque area growth (relative)	0.43 ± 0.19	0.31 ± 0.08	0.001
Total plaque area growth	0.67 ± 0.18	0.39 ± 0.07	< 0.001

Total plaque: visible plaque + non-visible plaque

SD, standard deviation

## Discussion

### Methodological approach

The efficacy of active chemical anti-plaque agents is usually assessed by quantifying dental plaque using conventional clinical indices. However, these measurements have several limitations that can produce inaccurate results and complicate agent comparisons [33, 34]. Consequently, to improve diagnoses of dental plaque, it is essential to employ computer systems that allow plaque levels to be determined objectively [35–37].

Our research team has recently developed DenTiUS Deep Plaque, a method that enables the assessment of the entire dentition. In particular, as well as discriminating between plaque and clean teeth, the software allows clinical indices to be obtained automatically [37]. The tool is based on a novel algorithm for detecting and quantifying dental plaque levels from ultraviolet images. The system identifies visible (mature plaque) and non-visible plaque (immature plaque that will soon become mature). Indices to quantify plaque and measure the plaque growth pattern over time can thus be calculated for both plaque types [37].

Although this is the first study in the literature to use our DenTiUS Deep Plaque image analysis software to evaluate the anti-plaque effect of a chemical agent, an internal validation conducted with an in situ 5-day bacterial plaque growth model found that the degree of correlation between the conventional (clinical) and the automated quantification indices was very high on days 1, 2, and 3 of plaque formation (Spearman rho ≥ 0.770) [41, 42]. Conversely, these relationships were suboptimal (Spearman rho ≤ 0.540) at the time points where there was little (day 0) or an excessive (day 4) accumulation of dental plaque, thus highlighting the limitations of the conventional approach and the convenience of employing the automated method made possible by DenTiUS Deep Plaque for these clinical situations [41, 42].

### Anti-plaque effect of cymenol

A meta-analysis and meta-regression study concluded that using EO-containing mouthwashes as adjuncts to mechanical plaque control is more effective at reducing plaque and gingival inflammation than brushing and flossing alone or combined with CPC rinses [43]. The present study evaluated the effect on dental plaque development in situ using a mouthwash containing 0.1% EO cymenol for eight consecutive days.

A literature review found that only a few studies to date have assessed the performance of cymenol for various purposes using in vitro [21–24] or in vivo [25–31] experiments, reflecting that this compound needs to be studied more.

About the in vitro studies, the majority have analysed aspects unrelated to the anti-plaque activity of cymenol, e.g., its capacity to reduce both the demineralisation of human enamel [23] and volatile sulphur compounds in halitosis models [21] and to be retained in reconstructed human gingival tissue [24]. In contrast, a further in vitro study used tubes of toothpaste containing cymenol and zinc at different concentrations (both alone and in combination) to evaluate their antimicrobial effect against some predefined oral pathogens [22]. These authors demonstrated that the cymenol/zinc system has direct anti-microbial effects and inhibits oral disease-related processes like glycolysis and protease activity [22].

Similarly, some in vivo investigations have evaluated aspects unrelated to the anti-plaque activity of cymenol, such as its ability to reduce xerostomia [29] and control oral malodour [31]. Other studies have analysed its substantivity up to four hours after application [30] and its ability to reduce gingival bleeding in patients with gingivitis [27], albeit without recording any clinical measurements of dental plaque. On the other hand, to our knowledge, only three investigations have evaluated the anti-plaque effect of cymenol using the Turesky modification of the Quigley Hein (TQH) index [25, 26, 28, 32]. Specifically, in two of these studies, orally healthy subjects who used cymenol toothpaste were compared with those employing sodium fluoride products over 12 weeks [25, 26]; alternatively, in further research, gingivitis patients who used cymenol toothpaste and mouthwash were contrasted with their baseline over 42 days [28].

As already discussed in the methodological approach section, the present trial’s automated image analysis method for evaluating levels of dental plaque produces more accurate results than those obtained previously [25, 26, 28] using traditional clinical indices such as Turesky [32]. In our study, orally healthy subjects were randomly assigned to one of two groups: test group A received the cymenol rinse for eight days, and test group B was given a mouthwash without active ingredients for four days and then the cymenol rinse for four days thereafter. This two-group and two-phase design of the trial permitted us to make independent between-group and dependent within-group comparisons. Furthermore, in contrast to previous publications [25, 26, 28], our participants were not allowed to brush their teeth during the experiment, as we wanted to identify the effect of cymenol alone on plaque levels. This prohibition of mechanical plaque removal justifies the shorter duration of our trial.

In comparison to sodium fluoride dentifrices, the use of cymenol-based toothpaste by orally healthy individuals has been found to significantly lower TQH scores by 13.20% after six weeks (i.e., 42 days) [25] and by 20.60% [26] to 24.20% [25] after 12 weeks (i.e., 84 days). We observed significant differences between the two analysed groups in the present research. The visible plaque area in the cymenol volunteers from test group A was 11% and 20% lower after four and eight days, respectively, than in test group B. There were more significant differences in a shorter time interval, possibly due to our use of the DenTiUS Deep Plaque automated clinical indices, which are more accurate at detecting plaque zones than the human eye [37]. Additionally, it may be that the adjuvant use of cymenol by rinsing has more advantages than its use in dentifrices, as reported for CHX (reduction in plaque indices vs. control: rinse alone = up to 71%; toothpaste alone = up to 24%) [6].

On the other hand, compared to their baseline, gingivitis patients using both a cymenol-containing toothpaste and a cymenol-mouthwash have been found to experience significant reductions in their mean TQH values of 38% after seven and 14 days, and 41% after 42 days [28]. In our study, the prohibition of mechanical hygiene measures meant that the visible and total plaque areas increased steadily over the eight days of the experiment. Nonetheless, the plaque area growth indices calculated using the DenTiUS Deep Plaque software enabled us to see in each group (paired-group analysis) how the use of the cymenol rinse in the last four days of the study reduced the rate of growth of the visible plaque area by ~ 36% in test group A and ~ 29% in test group B; for the growth rate of total plaque area, these reductions were 43% and 41%, respectively. In test group A, if the placebo values on day 4 are taken as the reference point (independent-group analysis), this reduction was even more considerable, reaching 53% and 48%, respectively. Given these indices’ novel nature, we could not compare the values obtained here with others reported in the literature.

One of this project’s main limitations was that the size of the study group samples probably conditioned the non-detection of significant differences in the total plaque area percentages between the two groups in the first four days of application. Although the total plaque levels of test group A were about 6% lower, the effect size established for the calculated sample size was 0.08 (equivalent to a difference of 8%). In addition, including a third study group who had received the placebo mouthwash without any active ingredients in both phase 1 and phase 2 would have enabled us to determine the pattern of the plaque growth rate in the last four days of the experiment. Regarding the limitations associated with the imaging methodology applied, it should be noted that digital images are two-dimensional photographs of a three-dimensional environment. On the other hand, digital images of the palatal and lingual surfaces were not taken. However, in this regard, several authors have demonstrated through clinical indices that plaque values on buccal surfaces are similar to those found on palatal/lingual surfaces, evidencing that dental plaque on the buccal surface is representative of plaque on the palatal/lingual surface [44, 45].

The future perspective of clinical studies on the efficacy of oral hygiene techniques or products requires automated methods to analyse dental plaque levels using imaging. This would enable more objective comparisons to be made between different chemical adjuvants. Consequently, further research with computerised tools based on artificial intelligence is necessary to determine the best active ingredients in the fight against dental plaque.

## Conclusions

The 0.1% cymenol mouthwash has a short-term anti-plaque effect in situ, strongly conditioning the plaque growth rate, even in clinical situations with high accumulation levels.

## Data Availability

Data will be made available on a case-by-case basis, and additional information will be provided by contacting the corresponding author.
